# Validation of the Center of Applied Psychology Female Sexuality Questionnaire (CAPFS-Q)

**DOI:** 10.3390/jcm10122686

**Published:** 2021-06-18

**Authors:** Sonia Tirado-González, Antonio Navarro-Sánchez, Antonio Compañ-Rosique, Paloma Luri-Prieto, Jesús Rodríguez-Marín, Carlos J. Van-der Hofstadt-Román, María Berenguer Soler, Felipe Navarro-Cremades, Vicente F. Gil-Guillén, Ramón Navarro Ortiz, Angel L. Montejo, Virtudes Pérez-Jover

**Affiliations:** 1 Faculty of Psychology, Miguel Hernández University, 03202 Elche, Spain; sonia.tirado@umh.es (S.T.-G.); navarrosancheztony@gmail.com (A.N.-S.); cjvander@umh.es (C.J.V.-d.H.-R.); mariia_269@hotmail.com (M.B.S.); v.perez@umh.es (V.P.-J.); 2 San Juan University Hospital, Miguel Hernández University, Ctra N-332, s/n, 03550 Sant Joan d’Alacant, Spain; af.company@umh.es (A.C.-R.); palomalurip@hotmail.es (P.L.-P.); 3 School of Medicine, Miguel Hernández University, 03550 San Juan, Spain; rod.marin@umh.es (J.R.-M.); felipe.navarro@umh.es (F.N.-C.); Vte.Gil@gmail.com (V.F.G.-G.); 4 Torrevieja University Hospital, Carretera CV 95, s/n, 03186 Torrevieja, Spain; ray_hard_@hotmail.com; 5 Psychiatry Service, Clinical Hospital of the University of Salamanca, 37007 Salamanca, Spain; 6 Institute of Biomedical Research of Salamanca (IBSAL), Paseo San Vicente SN, 37007 Salamanca, Spain; 7 Nursing School, University of Salamanca, Av. Donantes de Sangre SN, 37007 Salamanca, Spain

**Keywords:** sexuality, female, questionnaire, validity, psychometry

## Abstract

Instruments for the measurement of human sexuality include self-report measures used to assess sexual functioning, but many of them have not yet been validated. The Center of Applied Psychology Female Sexual Questionnaire (CAPFS-Q) is an original self-report instrument. It has been developed for the study of sexuality in specific non-clinical populations, such as female university students of Medicine and other Health Sciences. The CAPFS-Q includes 26 items, organized as follows: sociodemographic and relevant data (four items); aspects of sexual relations with partner (five items); sexual practices (12 from 13 items); and dysfunctional aspects of sexual relations (four items). CAPFS-Q validity and reliability were examined in a sample of Spanish female university students of Health Sciences. Exploratory and confirmatory factor analysis (FA) showed a four-factor structure which explained 71.6% of the variance. This initial version of the CAPFS-Q is a reliable measure of women’s sexual behavior, with a dimensionality that replicates the initial theoretical content and with adequate indicators of internal consistency, validity, and test–retest reliability. It is easy to administer and to complete.

## 1. Introduction

For many, human sexuality is a main component of the person’s life [1,2]. Many aspects of daily life, such as physical health, well-being, and the quality of relationships influence sexual functioning in sexually healthy individuals [3]. Sex and sexual function are an integral part of human behavior [4]. Human sexuality is a basic force that can affect all aspects of life [4], being a universal part of life; and positive sex and sexual function are increasingly recognized as important indicators of positive health and quality of life [5]. Sexual satisfaction is, for many, a strong predictor of dyadic relationship satisfaction [6] and general personal well-being [7]. Sexuality is a human experience throughout life [8]. Sexual feelings, desires, and activities extend throughout the life cycle [4]. Sexuality is a complex and multifaceted dimension of human beings [9], whose experience is unique to each individual and must be seen in its entirety [1]. Female sexuality is complex and multidimensional [10]. It includes psychological, interpersonal [1,11,12], biological, spiritual [11], and sociocultural aspects [11,13]. The definitions, concepts, and constructs of human sexuality are not univocal, but multiple and heterogeneous with great cross-cultural variation [14], which affects the characteristics of the instruments designed for their description and measurement. The instruments for the measurement of human sexuality have been and are used both in samples of the general population [14] and community health studies [15], as well as of clinical and other determined populations, such as specific gender populations and university student populations [14,16,17,18,19]. Although there are several methods to assess sexual functioning, not all of them have been validated [14]. The content of a sexuality measurement instrument includes the items, their format and wording, the response options, and the administration and scoring procedures [20]. Some questionnaires include items of sexual behaviors of the person and their partner [16].

Instruments such as the Female Sexual Function Index (FSFI) [21], one of the most widely used measures of female sexual dysfunction [22], have been developed as a brief multidimensional self-report instrument to assess the key dimensions of sexual function in women [23]. Their items were written by experts, and they are brief and easy to use [22]. Some of them highlight several factors, such as the measurement of female sexual arousal [24,25], sexual desire [24], and cognitive mediators [25]. The CAPFS-Q was designed by an expert committee on human sexuality based on their professional experience and relevant scientific articles [17,18]. The study of female sexual function (FSF) through self-administered questionnaires is a difficult task because of the complex, sensitive and personal nature of the matter, and because the measures are subjective [26]. These instruments include diverse attitudes, cognitions, and behaviors related to sexuality, personal, couple, relational variables, and other multiple related factors, sexological and other, such as health and its components [26] and the adverse effects of medications on sexuality [14,26,27,28,29]. Cognitive variables related to sexuality [16,25] may vary between different cultures and geographic regions [30]. Disparities in sexual functioning between them can be explained by differences in sexual beliefs [30]. Some of these questionnaires are related to the growing interest in the development of new pharmacological treatments for sexual dysfunctions [31]. There are many populations in which sexual function has not been measured. More research is needed to design appropriate questionnaires for different populations [4]. Many of the measuring instruments [30,32,33,34,35,36,37,38,39] are aimed at specific populations, such as students of Medicine and other Health Sciences, whose researchers in general have developed original instruments for their study including the current self-administered questionnaire CAPFS-Q, whose preliminary partial psychometric characteristics were summarized in a congress paper published as an abstract [40]. There is an absence of uniformity in the sexological questionnaires regarding psychometric tests [4]. The development of most specific instruments has been based on clinical experience, review of the literature, and previous questionnaires [4,16]. The CAPFS-Q has the same foundation and seeks to become part of the growing group of validated instruments. Content experts as a team of scholars/academics and clinical psychologists/sexologists reviewed the items and evaluated the adequacy of the items [4,16].

Several reviews of self-administered sexological questionnaires have been carried out, highlighting relevant characteristics of a selection of them, such as their items, the time of completion, their particular use, their main characteristics, and their outstanding psychometric characteristics [41]. Most validated questionnaires available for the evaluation of female sexuality have a previous temporal reference, such as the previous month [7] or the previous three months [16] as the current instrument.

Questions about the content of the questionnaires can be grouped into three main types: factual, such as demographic, behavioral, and attitudinal [42]. Several questions of the three types [16] are included in the CAPFS-Q. This questionnaire was designed to be a valid assessment instrument for addressing selected behavioral and cognitive questions of female sexual function in a sample of young female Spanish university students. The purpose for developping the CAPFS-Q was to capture, in an original self-report instrument, selected questions of interest discussed by the female students in university courses on human sexuality and to know their responses to these questions. CAPFS-Q focusses on female sexual function and specific problems as sexual risks [18] and fears [17], and to a lesser extent, on female sexual dysfunction.

It was developed through a series of stages, including selection of the initial items and conceptual validation with a panel of expert consultants [17,21]. It was followed by a pilot study [16].

All the students were training in human sexuality in their university courses. They were fully informed in the classroom about the CAPFS-Q and discussed it before completing the questionnaire.

The CAPFS-Q aims to be a valid measurement instrument with the ultimate goal of improving training on human sexuality.

We have considered that some items of the CAPFS-Q include especially sensitive data that are of high personal and ethical/legal relevance. The ethical and scientific justification of this study includes at least three main factors: 1. The CAPFS-Q has been designed to improve the human sexuality teaching/learning process of participants as future health professionals; to be especially trained in this area for the benefit of their future patients. 2. We have paid special attention to its ethical concerns. 3. We used the PICO research criteria. Participants: female university students. Intervention: voluntary completion of the CAPSF-Q. Comparison: any. Outcome: the CAPSF-Q analysis of the 16 items concerning sexual practices and dysfunctional aspects of sexual relations.

## 2. Experimental Section

### 2.1. Materials and Methods

#### 2.1.1. Study Population

The aim of this study was to examine female sexuality in a population of female Health Science university students in Spain. Several qualitative methods were previously utilized. The results, together with a literature review and consultation with experts, were incorporated into the design and application of an original questionnaire called the Female Sexuality Questionnaire (CAPFS-Q) used to conduct quantitative research, first in a pilot sample in 2004 [16] and subsequently in several consecutive samples [17,18].

#### 2.1.2. Study Design and Participants

Our aim was to design and validate a self-administered questionnaire to assess three specific types of sexual experience, as well as various relevant beliefs related to the sexual lives of the participants and other associated factors, such as the use or non-use of contraception and the specific methods used [16]. A cross-sectional study was conducted using nonprobability convenience sampling of Spanish female undergraduate Health Science students at Miguel Hernández University [17,18]. The study was undertaken between February 2005 and February 2009. As no variable varied over time (*p* > 0.05), time was not used as an explanatory variable (17,18).

#### 2.1.3. Variables and Measurements

This article describes the validation of the CAPFS questionnaire (CAPFS-Q) designed by the Center for Applied Psychology of Miguel Hernández University. This questionnaire contains 26 items and was administered in the classroom in a sample of about 500 female undergraduate Health Science students aged from 19 to 23 years, with a grouping of those aged 24 or more years. The following types of sexual activity were the main focus of our study: self-masturbation, non-coital sexual relations, and vaginal intercourse, together with certain self-reported characteristics for each of them: frequency of activity, arousal, satisfaction, and frequency of orgasm. 

#### 2.1.4. Sample Size

A total of 601 students were invited to participate in the study. Of these students, nine declined participation and did not complete the questionnaire, leaving the classroom, and 15 were excluded due to absence of inclusion criteria because they had not had intercourse in the previous 3 months. Seven students did not submit the questionnaire, and five left it completely blank. Consequently, a total of 565 questionnaires were completed [17,18], 492 of which were considered in the present analysis.

#### 2.1.5. Purpose of the Study

The objective of the present study [42,43,44] was to examine certain psychometric properties of the current version of the CAPFS-Q in a sample of female university students. To do this, the metric properties of the items comprising the scale were analyzed, and the factor structure was examined using exploratory factor analysis (EFA), which was contrasted by confirmatory factor analysis (CFA). After confirming the structure, the internal consistency reliability was analyzed, as well as various validity indicators of its measures.

#### 2.1.6. Participants

Two exclusion criteria were applied to the completed questionnaires: omission of more than 10 percent of the responses to the questionnaire and a failure to respond truthfully, contradictions, or serious inconsistencies. The failure to respond truthfully was assessed by comparing responses which were incompatible with each other. After the initial filtering, the sample included 492 women, with a valid response rate of 87.1%. All the students completed the questionnaire in the classroom, in a self-administered manner. The average completion time was 25 min. The participation of the students was voluntary. No compensation was given for participation and there was no penalty for not participating in the study. Of the valid cases, 97% reported that they were heterosexual and 96% said that they had no previous diseases. A total of 92.3% were single, although 66.5% reported having a stable partner. The age range varied between 19 and 24 or more years (M = 20.73; SD = 2.39) (Table 1).

In order to explore and confirm the factor structure of the scale, the sample was randomly divided into two subsamples (N_1_ = 244 and N_2_ = 248), with no significant differences between them in age (*t*_(490)_ = −0.415, *p* = 0.68), marital status (*χ*^2^_(2)_ = 2.95, *p* = 0.23), stable partner (*χ*^2^_(1)_ = 1.24, *p* = 0.27), sexual orientation (*χ*^2^_(2)_ = 4.84, *p* = 0.09), or contraceptive method (*χ*^2^_(8)_ = 3.76, *p* = 0.88). They can therefore be considered equivalent groups.

#### 2.1.7. Instrument

The CAPFS-Q current version [16,17,18] (Appendix B) comprising 26 items, was organized as follows:

- Sociodemographic and relevant data (four of the five items in the general questions):
  − Age, marital status, stable partner.
  − Usual contraceptive method.
- Aspects of sexual relations with partner (five items):
  − Sexual orientation.
  − Means through which orgasms take place.
  − Aspects of sex life respondent would like to change.
  − Aspects of sexual relations respondent considers unpleasant.
  − Aspects of sexual relations respondent thinks her partner considers unpleasant.
- Sexual practices (from 13 items, 12 were analyzed): masturbation, sexual relations without intercourse (no vaginal penetration), and sexual intercourse. The following aspects (12 items) were evaluated for each item:
  − Frequency (response scale from 1 = Never to 7 = 5–7 times per week).
  − Arousal when engaging in this activity (response scale from 0 = Low to 7 = High).
  − Satisfaction after engaging in this activity (response scale from 0 = Dissatisfied to 7 = Satisfied).
  − Frequency of orgasms with this activity (response scale from 0 = Never to 7 = Always).
- Dysfunctional aspects of sexual relations (four items):
  − Sex drive (response scale from 1 = Low to 7 = High).
  − Refusal to have sex when partner wants to and partner’s reaction (response scale from 1 = Very frequently to 5 = Never).
  − Comfort/discomfort with being naked in front of partner (response scale from 1 = Uncomfortable to 7 = Comfortable).
  − How satisfied respondent thinks partner is with sexual relations (response scale from 0 = Dissatisfied to 7 = Satisfied).

The analysis of the instrument was performed with the 16 items concerning sexual practices (12 items) and dysfunctional aspects of sexual relations (four items).

#### 2.1.8. Statistical Analysis

SPSS 19.0/21 was used to obtain the metric properties of the items and for the exploratory factor analysis (EFA) in Subsample 1. The extraction of the number of factors was performed using the principal components method with Kaiser (λ > 1), and the rotation by direct Oblimin. The reliability of each subscale was assessed with Cronbach’s alpha coefficient. To confirm the structure obtained through the EFA in the subsample, a CFA with structural equations was performed using the AMOS 19.0 program. The model parameters were estimated using the maximum likelihood method. The indices considered to evaluate the fit of the models were the ratio between chi-square and degrees of freedom (*χ*^2^/gL, the Tucker–Lewis index (TLI) [45], and the root mean square error of approximation (RMSEA) [46,47]. For the *χ*^2^/gL, ratio, values between 1 and 3 are considered good fit indices. TLI values between 0.90 and 0.95 are considered adequate, and above 0.95 are considered good. The RMSEA is regarded as the best indicator of overall fit [48]. For this indicator, values below 0.05 are considered optimal, although some authors [49] suggest that values between 0.05 and 0.08 indicate an acceptable fit, while values above 0.10 indicate a poor fit.

Finally, the analysis of validity focused on two perspectives. First, we analyzed the mean differences of the factors of the model between women with and without a stable partner, using Student’s *t*-test. Second, we analyzed the relationships between the factors in the model with Pearson’s zero-order correlation coefficient.

#### 2.1.9. Ethical Issues

This study was previously approved by the Miguel Hernández University Project Evaluation Office (Ethics Committee). The women who participated in this study verbally agreed and consented to collaborate voluntarily, freely, and anonymously [16,17,18]. This study was previously approved by the Miguel Hernández University Project Evaluation Office (Ethics Committee). The women who participated in this study verbally agreed and consented to collaborate voluntarily, freely, and anonymously [16,17,18]. Given the highly sensitive and intimate character of the CAPFS-Q content, special attention was paid to ethical issues during the academic course. All students were familiar and not distressed with the content of the questionnaire and their participation in it, evidenced by the voluntary and free decision of each participant on completing the questionnaire.

## 3. Results

### 3.1. Item Analysis and Internal Consistency

The results show that all response options were chosen for all items. As indicated in Table 2, the mean item responses range from 2.64 (item “How often do you masturbate”) to 5.97 (item “How satisfied do you think your partner is with your sexual relations”). The standard deviations range from 0.90 (item “Refuse to have sexual relations with your partner”) to 2.50 (item “How often do you have orgasms when you masturbate”). Thus, we can assume an adequate variability of scores.

All discrimination indices (corrected item-total correlations r_itc_) exceeded 0.30. However, the calculation of the Cronbach’s alpha value when eliminating each item indicated that the internal consistency of the factor “Relations without intercourse” increased when the item “Frequency of sexual relations without intercourse” was removed, leading to the decision to eliminate this item.

### 3.2. Exploratory Factor Analysis

The sample adequacy index (KMO = 0.806) and Bartlett’s test of sphericity (*χ*^2^_(105)_ = 2271.2; *p* = 0.000) indicated the suitability of the data for factor analysis. The extraction was performed by the principal components method using the Kaiser criterion (λ > 1), while the rotation method used was direct oblimin, which considers oblique factors (Table 3).

This results in four factors that explain 71.6% of the variance, as shown in Table 3. As can be seen, the factor structure obtained coincides exactly with the organization of the items in the questionnaire, including four factors: Masturbation, Relations without intercourse, and Relations with intercourse and Dysfunction.

### 3.3. Confirmatory Factor Analysis

From the classical approach, a sequential use of AFE and AFC has indeed been made, dividing the sample randomly into two sub-samples and exploring the underlying factor structure of the items in the first sample, and then trying to confirm that structure in the other half of the sample, as recommended by some authors, such as Brown [50].

However, the CFA may fail to confirm factor structures found in the EFAs, especially with related factors, as highlighted by different authors [51,52,53,54,55,56]. Therefore, two alternative four-factor, independent, and related models were tested. With respect to the one-factor model, it was tested to rule out unidimensionality.

− One-factor model that considers sexuality as an overall whole.
− Four related factors model (model B), which was the one found in the EFA of this study.
− Four-factor model (as in model B), but with independent factors (model C).

Table 4 displays the fit indices of the three models tested. The one-dimensionality (model A) of the scale was rejected, since none of the indices used reached the required threshold. As noted, the model with four related factors (model B) fits better than the model with four independent factors (model C). The ratio *χ*^2^/gL positions model B as the best fit of those tested, since it is the only one with a ratio lower than 3.

In the orthogonal model, the indices show the model fits the data poorly, since the relative *χ*^2^ index is greater than 3, and the TLI index falls below a value of 0.90, while the residual RMSEA is quite high. By contrast, the oblique model shows a good fit in all the indices.

**Figure A1:**
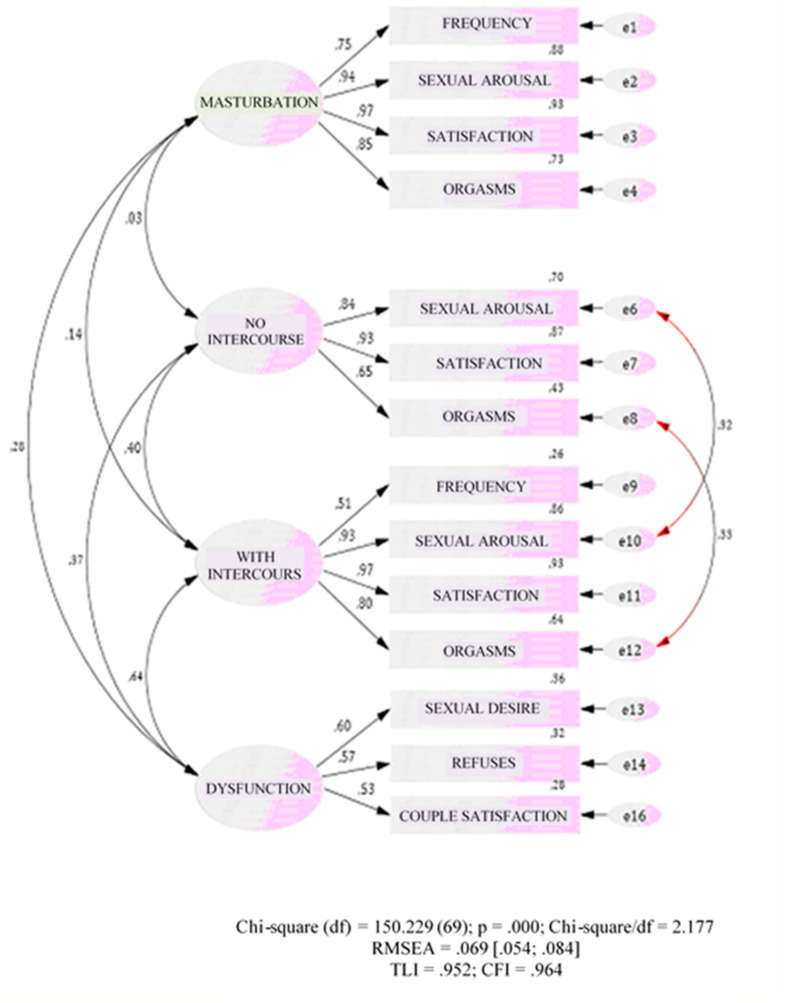
The path diagram. The model and the parameter estimates.

The model is represented in the path diagram in Figure A1, together with the parameter estimates. Again, the item “Nudity” performs slightly worse than the rest, with a standardized saturation of 0.53. However, as it is within the admissible values, the decision was made to retain it. Additionally, as suggested by the modification indices, the errors of the items “Sexual arousal in sexual relations without intercourse” and “Sexual arousal in sexual relations with intercourse”, on the one hand, and of the items “Achieving orgasms in sexual relations without intercourse” and “Achieving orgasms in sexual relations with intercourse”, on the other hand, were covaried.

### 3.4. Validity

Validity was tested by differentiating between groups. As indicated in Table 5, no significant or relevant differences exist between women with and without a stable partner in “Masturbation”, while such differences do exist in “Sexual relations without intercourse”, “Sexual relations with intercourse” and “Dysfunction”, in which women with a stable partner, on average, scored higher than women without a stable partner. The effect sizes are medium–high for the factors “Relations without intercourse” and “Dysfunction”, and medium–low for the factor “Relations without intercourse”.

In addition, the relationships found among the factors are presented in Table 6. As expected, the strongest associations were found among the factors “Sexual relations without intercourse”, “Sexual relations with intercourse”, and “Dysfunction”, while the factor “Masturbation” seems to be independent of the other types of sexual activity.

### 3.5. Test–Retest Reliability

In the CAPFS-Q test–retest study, the analyses carried out on the qualitative questions of the questionnaire did not show a relationship between the response of the participants and the group for any of the items, as the outcomes of all the two tests have been non-significant (Table A1 in the Appendix A). In the same way, the Student’s *t* test applied to the quantitative questions did not reveal any significant differences between the scores obtained in the test and the retest (Table A2 in Appendix A).

## 4. Discussion

In this study, several psychometric properties of the CAPFS-Q were examined in a sample of female university students studying Health Sciences. Having valid and reliable instruments to assess components of sexual health is of utmost importance [57]. Overall, the indicators obtained were good. In line with the recommendation of some authors [58], the mean scores of the questionnaire items are very similar to the theoretical mean, with standard deviations close to 1. The psychometric properties of the items are adequate, with no item obtaining a corrected item-total correlation lower than 0.30. Nevertheless, we decided to remove the item “Frequency of sexual relations without intercourse”, since it improved the internal consistency of its corresponding factor. The EFA showed a four-factor structure, in accordance with the initial organization of the items in the questionnaire, which explained 71.6% of the variance. This structure was confirmed by CFA, such that the model with four interrelated factors (Masturbation, Relations without intercourse, Relations with intercourse, and Dysfunction) showed the best fit among those tested. The reliability of the subscales is adequate, with coefficients for each of them of at least 0.60, with the factor of Masturbation being very high (α = 0.93). In the validity analysis, the results show a logical and expected relationship between the factors of the CAPFS-Q. Moreover, they reveal differences between women who have a stable partner and those who do not in the case of “Masturbation”, “Relations with intercourse”, and “Relations without intercourse”, but not in the case of “Dysfunction”.

In short, it can be concluded that this current version of the CAPFS-Q is a reliable measure of women’s sexual behavior, with a dimensionality that replicates the initial theoretical content and with adequate indicators of internal consistency and validity. However, although the results suggest that the CAPFS-Q is psychometrically sound, it must be noted that the data should not be regarded as definitive conclusions. The CAPFS-Q internal validity is privileged in this work compared to other types with regard to content and construct validity. Further studies with more ambitious objectives would be of interest, for example, calculating the factorial invariance of women of different ages or backgrounds (not only students), with the aim of replicating the equivalence of the dimensionality of the scale in other samples. It would also be very beneficial to obtain scales for detecting deficiencies in sexual relations, to propose different interventions according to needs. Finally, it would be of great relevance, once the validity and reliability of the scale has been confirmed in a normal population, to adapt and validate the scale in clinical populations or populations with risky sexual behaviors.

## 5. Limitations

The generalizability of the results [59] of many studies based on non-probability convenience samples, such as this one, could be adversely affected. It would be of research interest to apply this instrument in different samples from other populations, both in the general population and in specific populations, such as female university students from other universities and other non-clinical populations.

## 6. Conclusions

The CAPFS-Q is an original self-administered instrument consisting of 26 items, which has been psychometrically validated as a reliable, multidimensional instrument to assess female sexual behavior. It is specifically designed to assess 16 items referring to sexual practices (12 items) and dysfunctional aspects of sexual relations (four items), as well as other items of research interest, such as the use of contraceptive methods [16,17,18]. The CAPFS-Q is easy to administer and to complete, is reliable and valid, and is useful for the intended research objectives applied to our sample, especially for its implementation in improving teaching/learning processes in sexology in female students of Medicine and related Health Science degrees.

## Figures and Tables

**Table 1:** Sample distribution.

	*N*	%
**Age**		
19	97	19.7
20	242	49.2
21	71	14.4
22	22	4.5
23	26	5.3
24 or more	34	6.8
**Marital status**		
Single	454	92.3
Married	16	3.3
Other	22	4.5
**Sexual orientation**		
Bisexual	11	2.2
Heterosexual	477	97.0
Homosexual	4	0.8
**Contraceptive method**		
Condom	313	63.6
Hormonal contraceptive	89	18.1
None	31	6.3
Various	24	4.9
Vaginal ring	12	2.4
Withdrawal	6	1.2
Patches	4	0.8
IUD	1	0.2
**Stable partner**		
Yes	327	66.5
No	165	33.5

**Table 2:** Item analysis and internal consistency of factors.

Ítems	Mean	S.D.	ritc	Alfa-i
Masturbation (α = 0.93)				
Frequency	2.64	1.69	0.76	0.94
Excitement	4.30	2.25	0.90	0.89
Satisfaction	4.22	2.28	0.91	0.89
Orgasm Frequency	4.32	2.50	0.83	0.92
Relationships without intercourse (α = 0.801)				
Frequency	3.86	1.08	0.36	0.85
Excitement	5.59	1.32	0.76	0.68
Satisfaction	5.63	1.29	0.79	0.67
Orgasm Frequency	4.94	1.70	0.62	0.77
Relationships with intercourse (α = 0.842)				
Frequency	3.69	1.07	0.52	0.86
Excitement	5.61	1.44	0.77	0.76
Satisfaction	5.58	1.50	0.84	0.72
Orgasm Frequency	4.59	1.88	0.65	0.83
Dysfunction (α = 0.661)				
Appetite	5.17	1.20	0.42	0.61
Refuses	3.66	0.90	0.42	0.62
Couple satisfaction	5.97	1.18	0.56	0.51
Nakedness	5.49	1.60	0.43	0.63

**Table 3:** Item analysis and internal consistency of the factors. S^2^ explained.

Items	FI	FII	FIII	FIV	h2
Masturbation (α = 0.93)					
Frequency	−0.17	0.86	−0.06	0.09	0.78
Arousal	0.11	0.94	0.02	−0.03	0.90
Satisfaction	0.05	0.95	−0.04	0.02	0.91
Frequency of orgasms	0.07	0.90	0.10	−0.06	0.83
Relations without intercourse (α = 0.852)					
Arousal	0.01	−0.06	0.87	0.10	0.83
Satisfaction	−0.03	−0.01	0.89	0.11	0.84
Frequency of orgasms	0.02	0.07	0.87	−0.11	0.72
Relations with intercourse (α = 0.842)					
Frequency	0.66	0.13	−0.06	0.13	0.51
Arousal	0.81	−0.01	0.04	0.13	0.78
Satisfaction	0.92	0.00	0.02	−0.02	0.85
Frequency of orgasms	0.83	−0.05	0.06	−0.11	0.67
Dysfunction (α = 0.661)					
Appetite	−0.03	0.09	0.19	0.65	0.55
Refusal	−0.11	0.07	−0.06	0.81	0.60
Satisfaction, partner	0.18	−0.09	0.01	0.67	0.56
Nudity	0.22	−0.05	0.07	0.50	0.42
S^2^ explained	32.87	21.17	9.84	7.70	

**Table 4:** Goodness-of-fit indices for the CAPFS-Q.

Model	*χ* ^2^	gL	p	*χ*^2^/gL	TLI	RMSEA	IC RMSEA	p_RMSEA_
Model A One factor	1409.96	77	0.000	18.311	0.298	0.265	[0.253; 0.277]	0.000
Model B Four oblique factors	150.23	69	0.000	2.177	0.952	0.069	[0.054; 0.084]	0.020
Model C Four orthogonal factors	261.68	75	0.000	3.489	0.899	0.100	[0.087; 0.114]	0.000

**Table 5:** Differences in the means of the microsocial dimensions by group.

	With Partner		Without Partner		t_(242)_	δ	*r*
	Mean	S.D.	Mean	S.D.			
Masturbation	3.88	1.95	3.83	2.11	0.19 ns	0.03	0.01
Sexual relations without intercourse	5.21	0.86	4.62	1.21	4.32 **	0.60	0.27
Sexual relations with intercourse	4.99	1.11	4.60	1.44	2.29 *	0.32	0.15
Dysfunction	5.23	0.73	4.72	1.06	4.43 **	0.61	0.27

** *p* < 0.001; * *p* < 0.01: ns = not significant.

**Table 6:** Correlations among factors.

	Sexual Relations without Intercourse	Sexual Relations with Intercourse	Dysfunction
Masturbation	0.031 ns	0.138 ns	0.285 *
Sexual relations without intercourse		0.405 **	0.368 **
Sexual relations with intercourse			0.638 **
Dysfunction			

** *p* < 0.001; * *p* < 0.01; ns = no significant.

## Data Availability

The data that support the findings of this study are not publicly available due to reasons concerning privacy of the subjects and because there are personal sensible data under legal protection and restrictions from the European Union.

## Appendix A

Table A1 and Table A2; and Figure A1.

**Table A1:** Test–retest reliability. Descriptive and bivariant qualitative.

Variable	Categories	N (%) N = 117	N (%) Test N = 62	N (%) Retest N = 55	*p*-Value
Sexual Orientation	Bisexual	2 (1.7)	2 (100.0)	0 (0)	N.S.
	Heterosexual	115 (98.3)	60 (52.2)	55 (47.8)	
	Homosexual and	0 (0)	0 (0)	0 (0)	
	Other				
Use of condom?	No	53 (45.3)	28 (52.8)	25 (47.2)	N.S.
	Yes	64 (54.7)	34 (53.1)	30 (46.9)	
Stable relationship	No	72 (61.5)	37 (51.4)	35 (48.6)	N.S.
	Yes	45 (38.5)	25 (55.6)	20 (44.4)	
Masturbation	No	87 (74.4)	48 (55.2)	39 (44.8)	N.S.
	Yes	25 (21.4)	12 (48.0)	13 (52.0)	
To increase the frequency of sexual relations?	No	48 (41.0)	26 (54.2)	22 (45.8)	N.S.
	Yes	69 (59.0)	36 (52.2)	33 (47.8)	
To have the same sexual appetite as my partner	No	90 (76.9)	48 (53.3)	42 (46.7)	N.S.
	Yes	26 (22.2)	14 (53.8)	12 (46.2)	
To increase my capacity to have orgasms	No	75 (64.1)	40 (53.3)	35 (46.7)	N.S.
	Yes	42 (35.9)	22 (52.4)	20 (47.6)	
To eliminate the fear of pregnancy	No	80 (68.4)	41 (51.3)	39 (48.8)	N.S.
	Yes	37 (31.6)	21 (56.8)	16 (43.2)	
To increase my partner’s capacity to delay orgasm	No	102 (87.2)	54 (52.9)	48 (47.1)	N.S.
	Yes	15 (12.8)	8 (53.3)	7 (46.7)	
More variety	No	85 (72.6)	45 (52.9)	40 (47.1)	N.S.
	Yes	32 (27.4)	17 (53.1)	15 (46.9)	
Nothing	I would change something	110 (94.0)	58 (52.7)	52 (47.3)	N.S.
	I would not change anything	7 (6.0)	4 (57.1)	3 (42.9)	
Others	No	109 (93.2)	57 (52.3)	52 (47.7)	N.S.
	Yes	8 (6.8)	5 (62.5)	3 (37.5)	
Shows little enthusiasm	No	108 (92.3)	57 (52.8)	51 (47.2)	N.S.
	Yes	9 (7.7)	5 (55.6)	4 (44.4)	
Penis is too small	No	113 (96.6)	60 (53.1)	53 (46.9)	N.S.
	Yes	4 (3.4)	2 (50.0)	2 (50.0)	
Has difficulty maintaining an erection	No	113 (96.6)	60 (53.1)	53 (46.9)	N.S.
	Yes	4 (3.4)	2 (50.0)	2 (50.0)	
Too slow in ejaculating	No	116 (99.1)	62 (53.4)	54 (46.6)	N.S.
	Yes	1 (.9)	0 (0)	1 (100.0)	
Wishes to remove his penis too quickly	No	114 (97.4)	61 (53.5)	53 (46.5)	N.S.
	Yes	3 (2.6)	1 (33.3)	2 (66.7)	
Wants intercourse too frequently	No	97 (82.9)	51 (52.6)	46 (47.4)	N.S.
	Yes	19 (16.2)	11 (57.9)	8 (42.1)	
Penis is too big	No	115 (98.3)	61 (53.0)	54 (47.0)	N.S.
	Yes	2 (1.7)	1 (50.0)	1 (50.0)	
Cannot always ejaculate	No	114 (97.4)	60 (52.6)	54 (47.4)	N.S.
	Yes	3 (2.6)	2 (66.7)	1 (33.3)	
Ejaculates too quickly	No	97 (82.9)	52 (53.6)	45 (46.4)	N.S.
	Yes	20 (17.1)	10 (50.0)	10 (50.0)	
Wishes to sleep after intercourse	No	109 (93.2)	57 (52.3)	52 (47.7)	N.S.
	Yes	8 (6.8)	5 (62.5)	3 (37.5)	
Too sexually demanding	No	112 (95.7)	59 (52.7)	53 (47.3)	N.S.
	Yes	5 (4.3)	3 (60.0)	2 (40.0)	
Is not caring enough during intercourse	No	113 (96.6)	60 (53.1)	53 (46.9)	N.S.
	Yes	4 (3.4)	2 (50.0)	2 (50.0)	
Wants to do things that do not seem natural for me	No	110 (94.0)	59 (53.6)	51 (46.4)	N.S.
	Yes	6 (5.1)	3 (50.0)	3 (50.0)	
Cares little for my sexual satisfaction	No	111 (94.9)	58 (52.3)	53 (47.7)	N.S.
	Yes	6 (5.1)	4 (66.7)	2 (33.3)	
Does not stimulate or caress me enough before intercourse	No	101 (86.3)	53 (52.5)	48 (47.5)	N.S.
	Yes	16 (13.7)	9 (56.3)	7 (43.8)	
I show little enthusiasm	No	97 (82.9)	51 (52.6)	46 (47.4)	N.S.
	Yes	20 (17.1)	11 (55.0)	9 (45.0)	
Vagina is too small	No	115 (98.3)	61 (53.0)	54 (47.0)	N.S.
	Yes	2 (1.7)	1 (50.0)	1 (50.0)	
I never have an orgasm	No	115 (98.3)	61 (53.0)	54 (47.0)	N.S.
	Yes	2 (1.7)	1 (50.0)	1 (50.0)	
I orgasm too quickly	No	111 (94.9)	59 (53.2)	52 (46.8)	N.S.
	Yes	6 (5.1)	3 (50.0)	3 (50.0)	
I desire intercourse too frequently	No	114 (97.4)	60 (52.6)	54 (47.4)	N.S.
	Yes	2 (1.7)	2 (100.0)	0 (0)	
I like to practice unnatural things	No	114 (97.4)	61 (53.5)	53 (46.5)	N.S.
	Yes	3 (2.6)	1 (33.3)	2 (66.7)	
I am too slow at attaining an orgasm	No	85 (72.6)	44 (51.8)	41 (48.2)	N.S.
	Yes	31 (26.5)	18 (58.1)	13 (41.9)	
I want to sleep after I have had an orgasm	No	113 (96.6)	60 (53.1)	53 (46.9)	N.S.
	Yes	4 (3.4)	2 (50.0)	2 (50.0)	
I rarely want sex	No	106 (90.6)	57 (53.8)	49 (46.2)	N.S.
	Yes	11 (9.4)	5 (45.5)	6 (54.5)	
I do not stimulate and caress him enough before sex	No	107 (91.5)	55 (51.4)	52 (48.6)	N.S.
	Yes	10 (8.5)	7 (70.0)	3 (30.0)	

*p*-value. All differences are not significant. None are statistically significant.

**Table A2:** Test–retest reliability. Descriptive and bivariant quantitative.

Variable	$\bar{X}\left(\sigma \right)$ N = 117		$\bar{X}\left(\sigma \right)$ Test N = 62	$\bar{X}\left(\sigma \right)$ Retest N = 55	*p*-Value
Age	20.26	(0.66)	20.27 (0.66)	20.25 (0.67)	N.S.
Sexual appetite	5.16	(1.21)	5.19 (1.14)	5.13 (1.29)	N.S.
Masturbation frequency	4.25	(1.25)	4.17 (1.33)	4.35 (1.17)	N.S.
Masturbation excitation	4.81	(1.34)	4.73 (1.33)	4.90 (1.37)	N.S.
Masturbation satisfaction	4.86	(1.41)	4.90 (1.42)	4.83 (1.41)	N.S.
Masturbation orgasms frequency	5.45	(1.80)	5.35 (1.79)	5.58 (1.82)	N.S.
Non-vaginal intercourse with your partner frequency	3.75	(1.26)	3.73 (1.24)	3.76 (1.29)	N.S.
Non-vaginal intercourse with your partner excitation	5.88	(1.15)	5.87 (1.10)	5.89 (1.21)	N.S.
Non-vaginal intercourse with your partner satisfaction	5.76	(1.13)	5.73 (1.07)	5.80 (1.22)	N.S.
Non-vaginal intercourse with your partner orgasms frequency	5.19	(1.67)	5.08 (1.70)	5.31 (1.65)	N.S.
Vaginal intercourse with your partner frequency	3.48	(1.47)	3.55 (1.47)	3.40 (1.47)	N.S.
Vaginal intercourse with your partner excitation	5.85	(1.34)	5.86 (1.31)	5.84 (1.40)	N.S.
Vaginal intercourse with your partner satisfaction	5.63	(1.31)	5.60 (1.29)	5.66 (1.35)	N.S.
Vaginal intercourse with your partner orgasms frequency	4.65	(1.84)	4.66 (1.81)	4.64 (1.90)	N.S.
How do you feel when you are naked in front of your partner?	5.61	(1.63)	5.57 (1.63)	5.65 (1.64)	N.S.
What do you believe is your partner’s level of satisfaction in your sexual relationship?	5.77	(1.30)	5.75 (1.37)	5.78 (1.23)	N.S.

*p*-value. All differences are not significant. None are statistically significant.

## Appendix B

The Center of Applied Psychology Female Sexuality Questionnaire (CAPFS-Q).

General Questions
A Age: 19, 20, 21, 22, 23, 24 or more B Marital status: Single; Married; Widow; Separated; Divorced; Other C Sexual orientation: Bisexual; Heterosexual; Homosexual; Other D Contraceptive method used: 1 condom, 2 oral contraceptive, 3 none, 4 withdrawal, 5 IUD, 6 vaginal ring, 7 patches, 8 various, 9 abstinence. E Do you have a stable partner? Yes No
The following questions refer to the last 3 months.
General sexuality items
1. Suppose the following is a scale of SEX DRIVE. Where would you place yourself on this scale? Low 1 2 3 4 5 6 7 High.
Masturbation items
2. Have you ever masturbated? Yes No 3. How often? 5–7 times/week 3–4 times/week 1–2 times/week 2–3 times/month Once a month Less than once a month 4. Suppose the following is a sexual AROUSAL scale. Where would you place yourself on this scale when engaging in this type of sexual activity? Low 1 2 3 4 5 6 7 High. 5. How satisfied are you with this type of sexual activity? Dissatisfied 1 2 3 4 5 6 7 Satisfied 6. How often do you have orgasms with this type of sexual activity? Never 1 2 3 4 5 6 7 Always
Non-vaginal sexual relations items
7. How often do you have sexual relations with your partner, excluding sexual intercourse? (Including manual stimulation, oral stimulation, etc.)? 5–7 times/week 3–4 times/week 1–2 times/week 2–3 times/month Once a month Never 8. Suppose the following is a scale of sexual AROUSAL. Where would you place yourself when engaging in this type of sexual activity? Low 1 2 3 4 5 6 7 High 9. How satisfied are you with this type of sexual activity? Dissatisfied 1 2 3 4 5 6 7 Satisfied 10. How often do you have orgasms during this type of sexual activity? Never 1 2 3 4 5 6 7 Always
Vaginal intercourse items
11. How often do you have vaginal intercourse with your partner? 5–7 times/week 3–4 times/week 1–2 times/week 2–3 times/month Once a month Never 12. Suppose the following is a scale of sexual AROUSAL. Where would you place yourself when engaging in this type of sexual activity? Low 1 2 3 4 5 6 7 High 13. How satisfied are you with this type of sexual activity? Dissatisfied 1 2 3 4 5 6 7 Satisfied 14. How often do you have orgasms while engaging in this type of sexual activity? Never 1 2 3 4 5 6 7 Always
Orgasm items
15. How do your orgasms usually take place? During vaginal intercourse Through fantasies and daydreams Through stimulation by my partner Through self-stimulation Through several of the above methods I do not have orgasms By other methods
Items concerning changes in sex life
16. What aspects of your sex life would you change? Increase the frequency of sexual relations Have the same sex drive as my partner Improve my ability to have orgasms Eliminate fear of pregnancy Increase my partner’s ability to delay orgasm. Increased variety (time of day, position, etc.) None Other__Which?
Sexual partner items
17. Regarding your partner, indicate the things that you find unpleasant in your sexual relations: He shows little enthusiasm Penis too small He has difficulty maintaining an erection Too slow to ejaculate He wants to withdraw his penis too quickly He wants intercourse too often Penis too large He has difficulty achieving erection He cannot always ejaculate He ejaculates quickly He wants to sleep after intercourse He rarely wants intercourse He is too sexually demanding Is very unaffectionate during intercourse He wants to do things that do not seem natural to me Cares little about my sexual satisfaction He does not stimulate or caress me enough before intercourse 18. Do you refuse to have intimate sexual relations when your partner wants you to? Very often Frequently Sometimes Rarely Never 19. If you refuse to have sexual relations with your partner, how does your partner react? Insistent or irritable Annoyed, but not for very long Considerate and pleasant 20. How do you feel when you are naked in front of your partner? UncomforTable 1 2 3 4 5 6 7 Comfortable 21. How satisfied do you think your partner is with your sexual relations? Dissatisfied 1 2 3 4 5 6 7 Very satisfied 22. With regard to yourself, what things do you think your partner finds unpleasant in your sexual relations? I show little enthusiasm Vagina too small I never have an orgasm I have an orgasm too quickly I want intercourse too often I am not very affectionate during intercourse I like to do unnatural things Vagina too big I am too slow to have an orgasm I want to sleep after having an orgasm I rarely want intercourse I do not stimulate and caress my partner sufficiently before intercourse
